# Eyes of love: Java sparrows increase eye ring conspicuousness when pair-bonded

**DOI:** 10.1371/journal.pone.0292074

**Published:** 2023-10-25

**Authors:** Jenna Onaga, Masayo Soma

**Affiliations:** 1 Biosystems Science Course, Graduate School of Life Science, Hokkaido University, Sapporo, Japan; 2 Department of Biology, Faculty of Science, Hokkaido University, Sapporo, Japan; University of Illinois Chicago Department of Biological Sciences, UNITED STATES

## Abstract

Conspicuous facial features, such as blushing in primates, can communicate social/emotional/physiological states in animals. However, the role of bare facial features is less well studied in birds than in humans or primates. We investigate the Java sparrow, which is characterised by conspicuous rings of swollen and blushed bare skin around the eye. Eye rings show no clear sex difference, although the swelling is associated with breeding. Java sparrows are socially monogamous, with mutual courtships and long-term pair-bonding. Therefore, it is plausible that eye rings function in within-pair communication. Specifically, do eye rings reflect psychophysiological conditions after pair formation? We assessed variations in ring thickness in pair-bonded birds and compared them with single birds and pairs of non-bonded individuals. Over the 12-week experimental period, pair-bonded males and females had an increased ring thickness, unlike the controls. We suggest eye rings convey breeding motivations or serve as fertility signals. This would be of great importance for ensuring reproductive synchrony in tropical birds like the Java sparrow. Our results contribute to understanding the evolution of facial ornamentation in birds, which was often overlooked in the past studies.

## Introduction

Bare skin colouration in terrestrial endothermic animals potentially plays an important role in conveying physical and emotional states of individuals through flushing or changes in pigment depositions [1, 2] (see also [3]). One of the most familiar examples in humans is blushing, which was previously discussed by Charles Darwin in “The Expression of the Emotions in Man and Animals” [4]. More recently, blushing in various other primates has attracted considerable attention, including investigating its proximate mechanisms and functions. A number of primate species are characterised by bare reddish facial skin, which is highly vascularised, reflecting blood flow (e.g. [5]), and is considered to provide social/sexual signals [6, 7]. For instance, rhesus macaque (*Macaca mulatta*) males with redder faces are more attractive to females [8]. Similarly in mandrills (*Mandrillus sphinx*), a red face is an indicator of social dominance in males [9] and age and fertility in females [10]. Despite accumulating research on primate facial appearances, there remains limited evidence that facial colouration includes short-term dynamic changes to communicate emotional states similar to humans [11].

Birds also exhibit colourful bare areas which may be used as signals, including legs, beaks, irises, pupils, and facial skin (including combs and wattles) [2, 12]. These areas are pigmented (with either carotenoids or melanin) or structurally coloured in a similar fashion to feathered sections, and a few have haemoglobin-based colourations [2, 13–16]. Pigmented and structural colouration have been widely examined as condition-dependent traits in plumage signalling [17–19]. However, the role of haemoglobin-based blushed colours in birds remains less understood. In the junglefowl (*Gallus gallus*), comb redness is thought to be haemoglobin-based and predicts sperm viability in males [20, 21], but its relative importance among multiple ornamental traits in the species remains controversial [21, 22]. A limited number of studies have described rapid flushing colours in birds in social contexts. In agonistic interactions, blushing might be associated with social dominance. For example, crested caracaras (*Caracara cheriway*) receiving aggression had a flushed cere but aggressors did not [23]. Conversely, lappet-faced vultures (*Aegypius tracheliotos*) with flushed heads were more likely to win an aggressive interaction [24]. In captive environments, blue and yellow macaws (*Ara ararauna*) showed increased blushing of bare cheek skin when they had positive social interactions with caretakers [25].

The eyes or eye areas in birds may exhibit stunning conspicuousness with signalling functions [2, 26]. For example, male Asities (family: Philepittidae) have bright blue/green-coloured wattles around the eyes that are absent in females [27], suggesting a role in sexual ornamentation. Iris colour changes with sex and/or age in several species [28, 29] (review in [30]). This implies that the eye area potentially conveys a large amount of visual information. A previous study on Japanese quails reported that pupil size increases with positive (i.e. rewarding) experiences [31]. Over the last half century, similar pupil size changes have been identified in humans and discussed in relation to communicative functions [32]. However, in birds, direct evidence of communication via dynamic eye morphology changes is scarce. Previous bird research is limited and mainly focuses on among-individual variations rather than within-individual changes.

**Fig 1:**
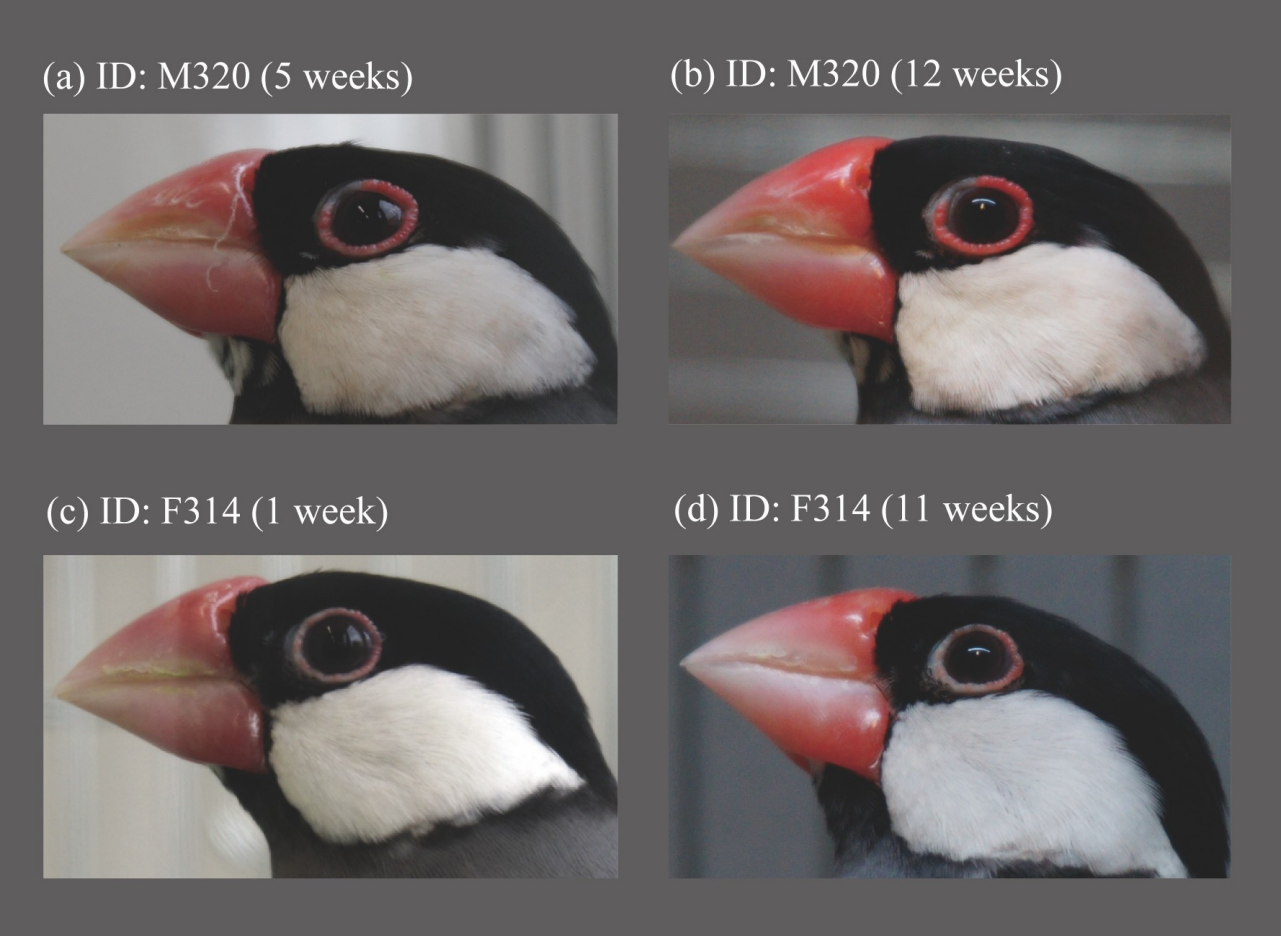
Eye ring changes in a male (a, b) and a female (c, d).

To understand the possible communicative functions of the bare skin surrounding bird eyes, we focus on the Java sparrow (*Lonchura oryzivora*), as the species has intriguing features. Both males and females have bright pink bare skin around the eye (Fig 1). These eye rings are said to swell when they are in breeding condition [33]. Just like other fleshy ornaments shown in birds [2], Java sparrow eye ring size appears to covary with redness. However, such size/color alterations have never been quantitatively assessed. We predict eye ring size changes reflect physiological conditions and signals breeding readiness. Such a signalling system would work especially well in species with long-term pair-bonds that breed opportunistically like the Java sparrow. Therefore, under the prediction that the eye ring would function as a signal especially between pair-bonded mating pairs, we compared within-individual changes in eye ring size between the individuals that were paired with preferred mate, those with a non-preferred opposite-sex individual, and single birds. We also examined sex differences in eye ring size to investigate possible differences in the roles of signalling traits between males and females.

## Methods

We used a total of 44 adult Java sparrows (*Lonchura oryzivora*) from a laboratory population. Changes in eye ring size were observed over a 12-week experimental period. Individuals investigated include: pair-bonded (n = 24), single (n = 18), or birds with a non-preferred partner (n = 10). Eight birds were used in both the pair-bonded and non-preferred partner conditions. First, the Java sparrows were allowed to form mating pairs by placing them in two unisex cages which were side by side. We observed the social interactions exchanged between birds in both cages. Male and female pairs displaying courtship behaviours (such as singing and dancing directed toward the other sex) were isolated in separate cages and further observed over a few days (for details of courtship see: [34–40]). Pair formation was determined based on social behaviours specific to pair-bonded birds, including clumping, allopreening, and copulation [36]. Those birds that did not form pairs were used for non-preferred partner condition, as described later. Each pair of bonded birds (n = 12 pairs) was housed in a cage (43 × 37 × 41 cm) for 12 weeks. Single birds (male: n = 10, female: n = 8) were used as controls and kept individually in the same sized cages over the same period. All cages were kept in the same aviary room where the birds were able to interact with others outside their cages acoustically but not visually. At the completion of the 12-week period, all birds were returned to their original unisex cages. Simulating the situation that Java sparrows can have second brood per season, five months later, the experiment was repeated using the pair-bonded birds in a within-subject control experiment. Some of the birds were moulting and unable to be used in the experiment. Therefore, three males and five females from the previous pair-bonded experiment were reused and two new males were randomly selected to create five non-bonded pairs. Because single Java sparrows are less choosy and can easily establish long-term pair-bonds even with non-preferred partners, we could not randomize or reverse the order of bonded and non-bonded conditions within subjects. We placed a male and female (not showing any pair-bonding behaviour towards each other) in cages (43 × 37 × 41 cm) (non-bonded birds: n = 10 pairs). All non-bonded pairs were observed for 12 weeks. We confirmed no pairs exhibited signs of pair bonding during the experimental period.

All experiments were performed in a controlled environment suitable for Java sparrow breeding (at a temperature of 25 ± 3°C; humidity 30–60%; 12L:12D photoperiod). Birds were provided a finch seed mixture ad libitum, consisting of foxtail millet (coated with egg yolk), rice, water, shell grit, and green vegetables.

In all experiments, photographs of both sides of each bird’s face were taken every week during the 12-week experimental period. During the first week, photographs included a ruler in the frame to determine the length and height of the eye. Every subsequent photograph did not include a ruler as the calculated eye length from the initial images provided the scale due to eye length being a consistent morphological feature. ImageJ [41] was used to capture the eye ring area. Each eye ring was measured by manually tracing the bare skin of the eye rings in each image which was then corrected using the eye length and height obtained in the initial photographs involving the ruler. To obtain accurate data of the eye ring area, we used the three best photographs of each bird face every week to calculate an average area size of each individual. We also measured the body mass of each individual when obtaining the eye ring data to evaluate within-individual changes in body condition.

To investigate weekly alterations in bird eye ring size (of the pair-bonded, single, or paired with non-preferred partner individuals) we used two linear mixed-effect (LME) models. We predicted the eye ring of pair-bonded birds may continue to increase in size or reach a peak during the observation period, and so considered the models with and without a quadratic term of week as an explanatory variable. We compared model fitting, relying on AIC. These models also incorporated sex as a fixed effect and bird identity as a random effect to address data dependence on information from the same individual. Similarly, we investigated the weekly body mass data.

This study was conducted with approval from the Institutional Animal Care and Use Committee of the National University Corporation at Hokkaido University (No. 16–0020) in accordance with the Hokkaido University Regulations of Animal Experimentation. During the study, stress was minimised, and all birds were cared for and treated appropriately in accordance with the Guidelines for Ethological Studies from the Japan Ethological Society.

## Results

**Fig 2:**
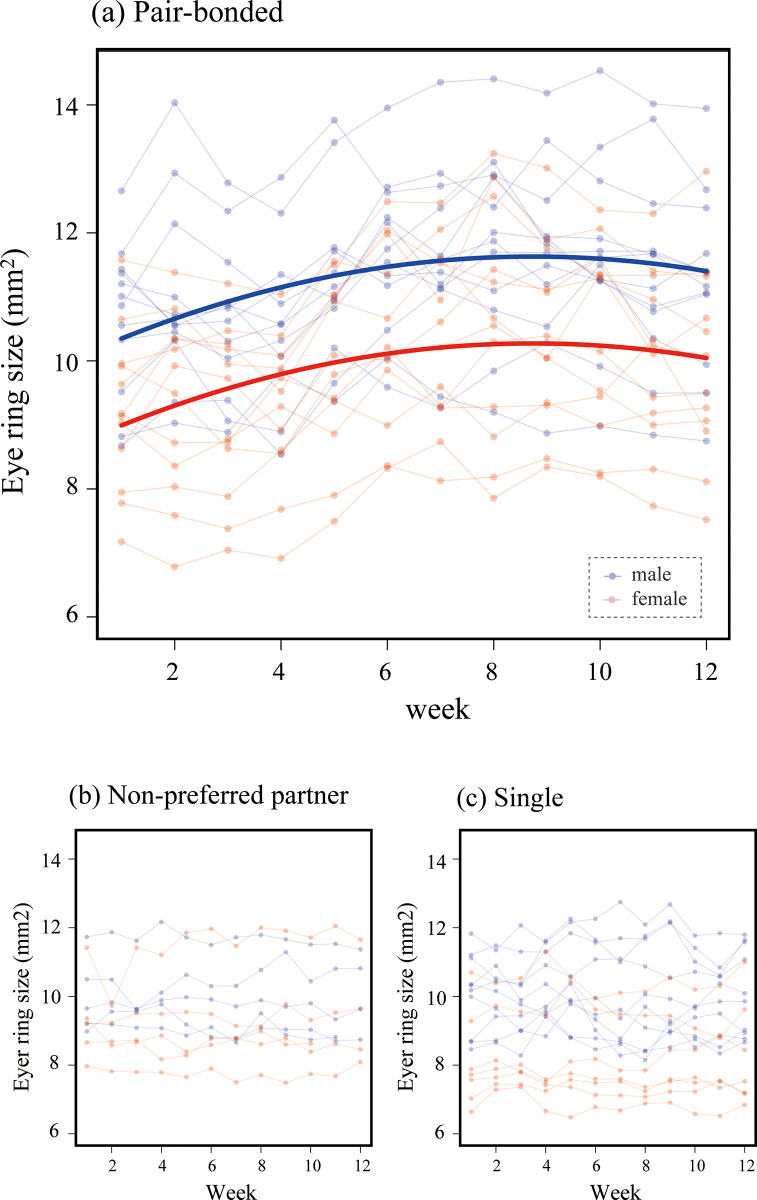
Weekly variations in eye ring size of the birds in the pair-bonded (a), non-preferred partner (b), and control single conditions (c). Blue and red connected dots represent male and female individuals, respectively. Bold lines provide the corresponding model estimates (Table 1a).

Eye rings in pair-bonded Java sparrows significantly increased in thickness over the duration of the experiment in both sexes (Fig 1). The model including the quadratic term of ‘week’ (AIC = 575.5) produced the best fit when compared to the model without the quadratic term (AIC = 612.3) and showed a statistically significant effect of the quadratic term of week on eye ring size (LME, week^2: p < 0.001; Table 1). Therefore, eye ring size plateaued during the 12-week observation period (Fig 2A). By contrast, eye ring size did not change significantly over time in the birds in the single or non-preferred partner conditions (Fig 2B and 2C), where the models without a quadratic term of week were selected based on AIC (Table 1). In both the pair-bonded and single conditions, males had larger eye rings, although this finding was not observed in the non-preferred mate conditions (Table 1).

**Table 1:** Weekly changes in eye ring area (mm^2^) (LME).

(a) Pair-bonded (with week^2, AIC = 575.5)												
Coefficient		SE	t	p								
Intercept	8.640	0.393	21.98	< 0.001								
**Week**	**0.374**	**0.039**	**9.54**	**< 0.001**								
**Week^2**	**-0.021**	**0.003**	**-7.29**	**< 0.001**								
**Sex (male)**	**1.357**	**0.535**	**2.54**	**0.019**								
(b) Pair-bonded (without week^2, AIC = 612.4)					(c) Non-preferred partner				(d) Single			
Coefficient		SE	t	p	Coefficient	SE	t	p	Coefficient	SE	t	p
Intercept	9.289	0.384	24.20	< 0.001	9.195	0.575	15.99	< 0.001	8.246	0.477	17.29	< 0.001
**Week**	**0.096**	**0.010**	**9.75**	**< 0.001**	0.002	0.009	0.20	0.841	-0.010	0.008	-1.23	0.221
**Sex (male)**	**1.357**	**0.535**	**2.54**	**0.0188**	0.804	0.809	0.99	0.350	**1.927**	**0.618**	**3.12**	**< 0.008**

Body mass varied differently from the eye ring results. Birds caged in pairs (in both conditions) gained weight during the 12-week observation period (bonded: p < 0.059, non-preferred: p < 0.005). Conversely, individually caged birds tended to lose weight (p < 0.086) (electronic S1 Table). All these eye ring and body mass data are available in S1 Data.

## Discussion

Eye rings of both male and female Java sparrows increased in size and thus potentially conspicuousness over the experimental period when they were with pair-bonded partners but not when kept alone or with a non-preferred partner. This finding supports our prediction that morphological signalling traits function after pair formation to convey breeding readiness. A review of the possible functions of song duets suggests duetting ensures reproductive synchrony within pairs, especially in the tropics where seasonal cues are absent [42] (see also [43]). This explanation can be applied to morphological traits which reflected physiological changes in breeding pairs. Indeed, Java sparrows are native to the tropics and bred most of the year (August to March in Thailand, [44]), wherein eye ring changes may signal mating readiness.

The present study highlights the role of brightly coloured facial bare skin areas in both sexes (Table 2). Small skin areas such as eye rings are not suitable for spectrometric analysis, which should be one of the reasons why avian bare skin ornamentation colour has been less well investigated than plumage-based colouration. Even so, by using standardized images we would be able to trace color changes as well. Similar to plumage, colourful bare areas on birds are thought to convey information on the quality of an individual for mate choice or mating competition [2]. A couple of previous studies on carotenoid-dependent eye rings in birds reported that their colourations reflects among-individual variations in body conditions [45, 46]. However, bare areas have the potential to change more dynamically or rapidly than plumage, which requires moulting to change (e.g. [25, 31, 47], Table 2). The eye ring in the Java sparrow can also be considered a condition indicator. Although the patterns of change in eye ring size differ from those of change in body mass, conspicuous eye rings can potentially serve as a fertility signal, similar to sexual swelling (with enlarged genital skin occurring in the receptive period) of female primates [48–50] (see [51] for similar morphological changes in an avian species). However, no other report on fertility signals shared between sexes exist (review in [52]). Java sparrows are known for long-term pair bonding and are characterised by a number of sexual signals shared between sexes which are used in within-pair communication (e.g. duet dancing [39] and vocal and non-vocal sound communication [36, 37, 53]), and eye rings can also be included in this list.

**Table 2:** Summary of signalling functions and possible mechanisms of colorful bare parts in birds.

	Flexibility to change				Factors associated with changes		
Signal function	Instantaneous or rapid (~ hours)	Mid-term (days, weeks)	Seasonal	Long-term stable	Sex	Honest indicator of individual quality	References
Emotional signal	✔				-	-	[20, 22, 27]
Sexual (mating) signal		✔ (this study)	✔	✔	Possible	Possible	[18, 19, 25, 26]
Social status signal	✔?	✔?	✔	✔	-	Possible	[21]
**Mechanism of conspicuousness**							
Flushing	✔	✔ (this study)			Possible	Possible	[13, 20–22]
Size		✔ (this study)	✔	✔	Possible	Possible	[18]
Pigmentation Structural color			✔	✔	Possible	Possible	[23–25, 41, 42]

According to a previous avian-wide comparative study [15], skin colour (i.e. black) is not sexually dimorphic in many species, which is in concord with our finding that Java sparrows did not show marked sexual dimorphism in eye rings. It should be noted that the lack of sexual dichromatism does not necessarily mean the absence of sexual selection. Probably, mutual sexual selection plays a role in some species like the Java sparrow. Interestingly, we observed that males have larger eye rings in the pair-bonded and single conditions, but not in the non-preferred partner condition. This can be interpreted as that males suppressed the expression of sexual signals when the situation was not favourable. Alternatively, it is also possible that males and females did not form pairs when they had similar eye ring sizes. However, non-significant sex difference in eye ring size could have been caused by one female that had relatively large eye rings (Fig 2B).

Further research is required to clarify the proximate mechanism of eye ring changes and the effect of experimental manipulation of the eye ring on a bonded Java sparrow partner. More importantly, it remains unclear why some species have conspicuous eyes or facial features, whereas others do not. The high diversity in facial appearance is known to play a role in individual recognition [54], and could also be an important sexual signal. Presumably, eye ring dynamics are subject to multiple selection pressures caused by sociality or breeding ecology in the species, which should be compared across other related species.

## Supporting information

S1 Table Body mass (g) changes in relation to week and sex (LME). (PDF)

S1 Data Data used in this study. (XLSX)
