# Supplementary material for: Eyes of love: Java sparrows increase eye ring conspicuousness when pair-bonded
Source: PLoS One. 2023 Oct 25;18(10):e0292074. doi: 10.1371/journal.pone.0292074 (PMC10599526; doi:10.1371/journal.pone.0292074)
Supplement: S1 Table — (PDF) [file pone.0292074.s001.pdf]

Electronic supplementary material

Table S1. Body mass (g) changes in relation to week and sex (LME).

	(b) Pair-bonded				(c) Non-preferred partner				(d) Single			
	Coefficient	SE	t	p	Coefficient	SE	t	p	Coefficient	SE	t	p
Intercept	28.471	0.642	44.34	< 0.001	26.340	0.910	28.94	< 0.001	26.824	0.721	37.21	< 0.001
**Week**	**0.025**	**0.013**	**1.90**	**0.059**	**0.067**	**0.023**	**2.90**	**0.005**	**-0.026**	**0.015**	**-1.73**	**0.086**
**Sex (male)**	0.202	0.900	0.22	0.824	2.199	1.270	1.73	0.122	1.759	0.931	1.89	0.078
